# The Perimenopausal Fatigue Self-Management Scale Is Suitable for Evaluating Perimenopausal Taiwanese Women’s Vulnerability to Fatigue Syndrome

**DOI:** 10.3390/healthcare9030336

**Published:** 2021-03-16

**Authors:** Hsiao-Hui Chiu, Lee-Ing Tsao, Chieh-Yu Liu, Yu-Ying Lu, Whei-Mei Shih, Peng-Hui Wang

**Affiliations:** 1 Department of Nursing, Taipei Veterans General Hospital, Taipei 112, Taiwan; shchiu2@vghtpe.gov.tw; 2 Graduate Institute of Gerontology and Heath Care Management, National Taipei University of Nursing and Health Sciences, Taipei 112, Taiwan; leeing.tsao@gmail.com (L.-I.T.); chiehyu@ntunhs.edu.tw (C.-Y.L.); yuyin@ntunhs.edu.tw (Y.-Y.L.); 3 Graduate Institute of Gerontology and Heath Care Management, Chang Gung University of Science and Technology, Taoyuan City 333, Taiwan; jeanshih@gw.cgust.edu.tw; 4 Department of Obstetrics and Gynecology, Taipei Veterans General Hospital, Taipei 112, Taiwan; 5 Institute of Clinical Medicine, National Yang Ming Chiao Tung University, Taipei 112, Taiwan; 6 Department of Medical Research, China Medical University Hospital, Taichung 404, Taiwan; 7 Female Cancer Foundation, Taipei 112, Taiwan

**Keywords:** confirmatory factor analysis, fatigue, menopause, postmenopausal, self-management scale

## Abstract

The purpose of this study was to test the feasibility of utilizing the established perimenopausal fatigue self-management scale (P-MFSMS) to evaluate perimenopausal Taiwanese women’s vulnerability to fatigue syndrome. A cross-sectional study design was adopted to survey 220 perimenopausal Taiwanese women with a mean age of 51.8 ± 4.64 years and a mean body mass index of 23.07 ± 3.04 kg/m^2^, 75.9% of whom were married, 52.3% had a college education or above, 80.4% had salaries, 81.3% had small families, and 96.4% were not using hormone therapy. The P-MFSMS consists of 25 questions based on six categories: (1) strive to maintain work energy and efficiency; (2) seek self-help from medical resources (doctor shopping); (3) strive to maintain the normal operation of the family (seeking help and support from family or significant other); (4) make time for activities or exercise in busy life; (5) slow down or adjust lifestyle; (6) frustration. For all of these six categories, the minimum loading of each question on the factor was calculated to be over 0.50, with a Cronbach’s α of 0.78 and a corrected total-item correlation of >0.50. The goodness of fit of the model was determined to be acceptable, with a chi-square/df value of <3.0 (*χ*^2^ = 503.45 and df = 260), a root mean square error of approximation (RMSEA) value of 0.065 (<0.08), as well as a Kaiser–Meyer–Olkin (KMO) value of 0.892. The Tucker–Lewis index (TLI = 0.91), Comparative Fit index (CFI = 0.92), and Incremental Fit index (IFI = 0.92) were all >0.90. There was no statistically significant difference in the difficulty between perimenopausal and postmenopausal women utilizing differential item function (DIF) analysis. Taken together, the 25-question P-MFSMS may be a potentially valid and reliable instrument for suitably evaluating perimenopausal Taiwanese women’s vulnerability to fatigue syndrome. Future studies will be conducted to test the effectiveness of the P-MFSMS for evaluating perimenopausal Taiwanese women’s vulnerability to fatigue syndrome in clinical practice.

## 1. Introduction

Fatigue, especially chronic fatigue syndrome (CFS), described as a feeling of lack of energy, weariness, loss of drive, decrease or loss of ability to sustain even routine activities, overwhelming feeling of tiredness, exhaustion, and physical or mental strain that occurs without conspicuous effort, is a debilitating and complex illness, accounting for sizable economic costs to individuals and contributing to a global problem as a whole [1,2,3,4]. Fatigue frequently occurs in women more so than men, and the prevalence of fatigue in menopausal women is 67.9%, which is significantly higher than that of women during the pre- and perimenopausal period [1,5]. Fatigue affects multiple body organs and functions, influencing both the physical and psychological systems [1].

Fatigue may also be considered a symptom or sign of perimenopausal status in women. However, sometimes, it is hard to distinguish fatigue from other symptoms or signs during menopausal transition (perimenopause). In fact, fatigue is commonly accompanied by other perimenopausal symptoms or signs, which also interact with one another to enter a vicious circle [2,6]. The common associated symptoms or signs include vasomotor symptoms (cold sweating, hot flashes on the face, and palpitation), physical problems (bone soreness and pain, backache, and numbness of the hands, feet, or skin), and psychological problems (feeling tired, absence of energy, anxiety, depression, distress, memory impairment (forgetfulness), insomnia, decreased libido, inability to concentrate, etc.) [2,3,4,5,6,7,8,9,10,11,12,13]. Among the aforementioned symptoms or signs, forgetfulness, insomnia, muscular and bone pain, and loss of energy are frequently reported in many studies [9,10,11,12,13]. Although there are so many symptoms or signs in women with fatigue, an estimated one-quarter of these women attempt to ask for an expert’s help [13]. Why do these symptomatic women not look for a health provider’s help? Limited access or a lack of awareness might be the main cause for this gap [14].

To overcome these barriers, providing health education to both health providers and patients may be a promising way to deliver respective supportive treatments [14]. In fact, symptom-awareness model campaigns have increasingly formed part of global disease (cancer, menopause, fatigue, etc.) control strategies [15,16,17], because lack of awareness is the most critical barrier for both health providers and patients [14]. Patients not only easily underestimate their symptoms, but also often fail to bring these symptoms to their health provider’s awareness [17]. The overlooking of symptoms is frequently found in health providers, partly because of missing and failing to identify problems, and partly because of a lack of willingness to manage these problems [17]. All of these factors contribute to a delayed diagnosis of fatigue syndrome in perimenopausal women and, subsequently, an unsatisfactory therapeutic outcome. Without adequate support, many menopausal women prone to fatigue syndrome can find it to be debilitating in many aspects of their lives. One study has shown that a higher modified Kupperman menopausal index is significantly associated with healthcare-seeking behavior [13], suggesting that health providers should implement an effective evaluated tool to improve patients’ healthcare-seeking behavior.

Based on the above findings, establishing an effective symptom-awareness model to evaluate perimenopausal Taiwanese women who are vulnerable to fatigue syndrome may help in the identification of such women who need support from medical personnel. To achieve this goal, we conducted this project to test the feasibility of utilizing the established perimenopausal fatigue self-management scale (P-MFSMS) to evaluate perimenopausal Taiwanese women’s vulnerability to fatigue syndrome.

## 2. Materials and Methods

### 2.1. Design

This study was a pilot study with a cross-sectional study design to test the applicability of the established P-MFSMS for perimenopausal women in Taiwan. The enrollment period was from November 2019 to January 2020. This study complied with research ethics and was approved by the Institutional Review Board (IRB: 2019-10-005CC).

### 2.2. Subjects

The enrollment site was the obstetrics and gynecology clinic of a medical center in a northern Taiwan community. The inclusion criteria included: (1) women aged 42–58 years; (2) those who had non-disease fatigue for more than one month and who went to the obstetrics and gynecology clinic or women in the community due to perimenopausal symptoms; (3) those able to communicate in Mandarin. The exclusion criteria included: (1) patients with major diseases, cancer, or mental illness; (2) those who had been long-term bedridden; (3) those whose menopause was caused by oophorectomy (surgery-related menopause); (4) those with abnormal cognitive function, unable to read and respond to questions, or unable to sign the consent form. Informed consent was obtained from all subjects involved in the study.

### 2.3. Development of the Perimenopausal Fatigue Self-Management Scale (P-MFSMS)

**Figure 1:**
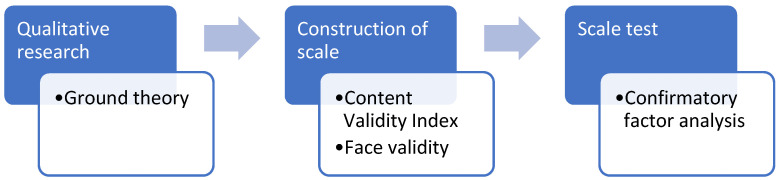
Flowchart of qualitative research.

The development of the P-MFSMS herein is summarized in Figure 1, which involved a total of three phases, staring from an initial exploration of the subject matter, encompassing the conceptualization of the ground theory of perimenopausal fatigue self-management, including the study of the backbone subject matter and the development of the specific items of the questionnaire; the following phase was the construction of the scale (the determination of the structure and format of the questionnaire and the assessment of content validity and face validity); the final phase was to test the scale by utilizing a quantitative approach, such as confirmatory factor analysis (CFA) or exploratory factor analysis (EFA), to conduct a pilot study.

In brief, 17 perimenopausal women in the obstetrics and gynecology clinic of a medical center in northern Taiwan were interviewed about their subjective fatigue experiences and the effectiveness of their fatigue management. The interview records were analyzed using grounded theory and then developed as six categories, as shown in Table 1.

Then, the developed 22 questions, including 20 closed questions and two open questions based on the aforementioned six categories, were selected using three methods for item selection (to eliminate indiscriminative items, to quantify the correlation between the score of each item and the total score to eliminate indiscriminative and low correlation coefficient items, and to further identify the principle component factor structure present in a set of variables by utilizing EFA with varimax rotation and CFA) [18,19,20]. An additional three questions were added due to the suggestions of five experts in different fields, including: (1) “You have found time to exercise to increase your physical fitness”; (2) “You have listened to music to relax”; (3) “You have meditated or sat still.” Five experts determined the items for content validity of the scale, and the evaluations of experts were scored using a content validity index (CVI). We used a Likert-type scale, ranging from 1 to 4. A score of one point meant the item was very unimportant, inappropriate, and unclear, and thus did not need to be included. A score of two points meant the item was unimportant, inappropriate, and unclear but needed to be corrected significantly. A score of three points meant the item was important, appropriate, and clear but required slight modification. A score of four points meant the item was very important, appropriate, and clear, and thus must be included (Table 2). The content validity showed that the CVI ranged from 0.82 to 1.00 for consistency, representativeness, relevance, and clarity of each construct, resulting in 25 items for the P-MFSMS. Perimenopause and menopause were based on the classification of the American Society for Reproductive Medicine’s Stages of Reproductive Aging Workshop (STRAW), which separates a woman’s life into seven segments, including segments −2, −1, and 0, ranging from the onset of menstrual cycles at menarche and the reproductive age to the peri-menopausal and postmenopausal phases [21,22,23]. In order to elucidate this distribution, women with regular menstrual bleeding during the last year were classified as premenopause; those with irregular bleeding during the last 12 months or with an age accompanied by less than one-year natural amenorrhea were defined as perimenopause; and the age at natural menopause was used to indicate the timing of menopause, which was confirmed after one year of amenorrhea [21,23]. Regular exercise was defined as either three or more sessions per week, for at least 20 min per session, of jogging–running, hiking, biking, swimming, or dancing resulting in a medium-to-large increase in reported heart rate or five or more sessions per week, for at least 30 min per session, of any physical activity, such as walking, gardening or yard work, or calisthenics, that resulted in at least some increase in reported heart rate [24].

### 2.4. Analysis

SPSS version 18.0 (SPSS Inc. released 2009. PASW Statistics for Windows, version 18.0. Chicago, IL, USA) and LISREL^®^ software version 8.8 (Scientific Software International, Inc., Skokie, IL, USA) were used for data analysis. Descriptive statistics were initially used to describe the sociodemographic profiles of all study subjects. CFA and EFA were used to test the applicability of the theoretical model [18,19,20,25,26,27,28].

## 3. Results

### 3.1. Demographics

A total of 220 subjects were enrolled with a mean age of 51.8 ± 4.64 years (mean ± standard deviation (SD)) and a mean body mass index (BMI) of 23.07 ± 3.04 kg/m^2^, 75.9% of whom were married, 52.3% had a college education or above, 80.4% had salaries, 81.3% had small families, and 96.4% were not using hormone therapy. For menstrual conditions, 60% of the subjects were perimenopausal, while 40% were menopausal. The mean age of menopause was 49 years old (SD = 4). Of the subjects, 64.1%, did not exercise regularly and 75.9% had no chronic diseases (Table 3).

### 3.2. The Perimenopausal Fatigue Self-Management Scale (P-MFSMS)

The feasibility of the 25-question P-MFSMS was evaluated by the abovementioned scoring system, ranging from 25% effective (one point) to 100% effective (four points). The mean range of scores was 1.7–2.4 points, which demonstrated that the degree of effectiveness ranged between 25% and 50%. The mean score of a single question was 2.1 ± 0.9 points (Table 4). The top three highest mean scores in order were “You have found partners to participate in activities” (2.4 ± 0.9), “You have found time to exercise to increase your physical fitness” (2.4 ± 1.0), and “You have listened to music to relax” (2.4 ± 1.0), which shows that “Make time for activities or exercise in busy life” was perceived as a relatively valid measure. The top three most commonly used management measures were “You can bear these fatigue experiences” (89.5%), “You have endured fatigue” (84.1%), and “You have listened to music to relax” (82.7%). Cronbach’s α was 0.78 and its corrected total-item correlation was >0.50, indicating that the P-MFSMS reached acceptable reliability [29,30,31]. There are different reports about the acceptable values of α, ranging from 0.70 to 0.95, and a low α appears if the assumptions of the essentially tau-equivalent approach are not met, but a high value of α (≥0.90) may suggest redundancies and show that the test length can be shortened [32,33,34,35,36,37,38,39,40].

The Kaiser–Meyer–Olkin (KMO) value in the current study was 0.892 and a KMO value of 0.80–0.89 is classified as good (meritorious) [41,42,43]. In theory, the KMO value was used to examine the measure of sampling adequacy (MSA), which ranged between 0 and 1, and the value more closely reached 1, suggesting that the correlation of the variables was much higher, and these variables were more suitable for factor analysis [27,41,42,43]. The correlation coefficient between the variables was *χ*^2^ = 503.45, df = 260, *p* < 0.001, suggesting that a significant Bartlett’s test of sphericity was found [44,45]. The principal factor analysis for the 25 items of the P-MFSMS was shown in RMSEA (root mean square error of approximation), and the result was 0.065. The value of the RMSEA in the current study was ≤0.08, indicating an adequate fit [41,46,47,48]. The Tucker–Lewis index (TLI) was 0.91. Both the Comparative Fit index (CFI) and the Incremental Fit index (IFI) were 0.92. Since an adequate fit of the TLI, CFI, and IFI is defined as a value >0.90 [41], the results of the fit indices in the current study all met the statistical requirements, suggesting that the P-MFSMS reached a satisfactory model fit.

Furthermore, the content validity of experts was used to present the degree of agreement of experts regarding the content of the measurement tool in a quantitative way, i.e., the CVI, which was scored by the importance, appropriateness, and clarity of the questions [49]. The four-point Likert-type scale was based on Soeken’s statement for validity of measures [49]. The CVI ranged from 0.82 to 1.0, suggesting that the items of the 25-question P-MFSMS were acceptable and appropriate for a factor analysis.

To further exclude the possibility of the presence of a biased assessment of group differences and confounding risk factors and outcomes in the current 25-question P-MFSMS, we performed differential item functioning (DIF) analysis [50,51,52] using Winsteps 4.4.3, and the results showed that there was no statistically significant difference in the difficulty between perimenopausal and postmenopausal women in the current study (Table 5).

**Figure 2:**
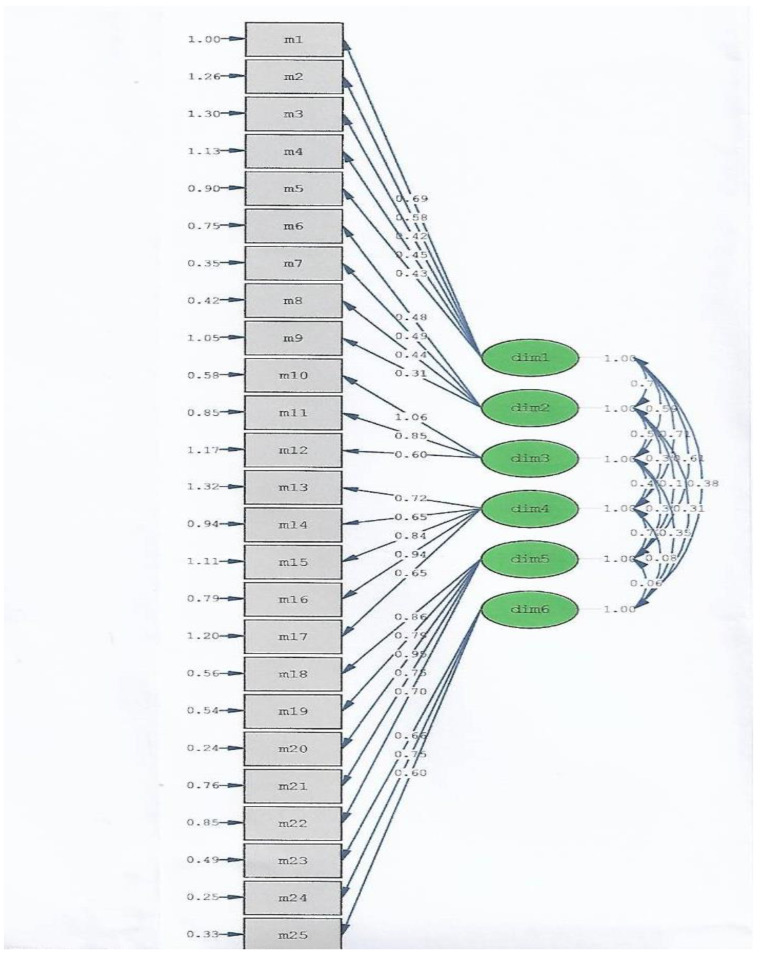
A confirmatory analysis of the P-MFSMS.

Taken together, the results indicate that the 25-question P-MFSMS can be applied to determine perimenopausal Taiwanese women’s vulnerability to fatigue syndrome by their value of the internal consistency indicator. The fit indexes indicate that the model was acceptable (Figure 2).

## 4. Discussion

The 25-question P-MFSMS was developed and validated as a new, self-administered instrument for the self-managing of fatigue in perimenopausal Taiwanese women, as shown in the current study.

Conventionally, CFA is used for construct validity tests. The dimension structure of the scale was confirmed in qualitative research by enrollment of subjects and calculation of various fitness indicators. The advantage of this research method is that it does not vary with different samples but emphasizes the value of “theory”. The best fit selection results of the CFA mode were the same as the theoretical framework of the design of this research questionnaire, which meant that the content of the questionnaire conformed to the theories found in qualitative research. In our study, the RMSEA was 0.065, which was ≤0.08, demonstrating that the current P-MFSMS model was acceptable. Moreover, the TLI was 0.91, and both the CFI and IFI were 0.92, showing that the results of the fit indices in the current study all met the statistical requirements.

Considering the possibility of the presence of biased assessment of group differences and confounding risk-factor and outcomes research in the current 25-question P-MFSMS based on the context of using multiple items to rate the level of a particular condition, where not all persons at the same level of the underlying condition (pre-menopause and post-menopause or age, as examples) had the same probability of endorsing one or more symptoms, DIF analysis should be performed [50,51,52]. For example, age, education level, self-management scale scores, and health literacy were significantly related to the health-related quality of life [52,53,54]. Our results showed that there was no statistically significant difference in the difficulty between peri-menopausal and menopausal women in the current study, indicating the feasibility of the current 25-question P-MFSMS for peri-menopausal and menopausal women prone to fatigue syndrome, even though the study subjects were not normally distributed in their background.

After in-depth interviews for qualitative research, the data had reached the saturation level for analysis, and the subjective experience of effective treatment methods for fatigue were summarized as 25 questions. The Cronbach’s α in the current study was 0.78, suggesting the reliability reached the acceptable level.

Our study found that the correlation between self-management for perimenopausal fatigue and perceived validity was satisfactory, since the commonly used management measures included enduring fatigue and listening to music; the 25% validity measures included feeling helpless, feeling angry, and asking family members to share the housework; the 50% validity measures included seeking healthcare attention, hormonal supplements, and sharing with others; the 75% validity measures included attending events, body massage, and keeping busy; the 100% validity measures were arranging activities or exercises. In fact, several studies have shown the possibility of benefits of fatigue self-management in clinical practice, including patients with cancer, neurological disease, and cardiovascular disease [53,54,55,56,57,58,59,60]. Studies have also shown that most behaviors are rated as providing moderate relief and are implemented with moderate self-efficacy in patients [55,56,57,58,59,60]. Yancey et al. suggested that encouraging timely rest, practicing relaxation techniques, cognitive behavioral therapy, and exercise could help women with fatigue syndrome to relieve their discomfort [59].

Several limitations of this study need to be noted. First of all, we randomly selected perimenopausal and menopausal women who claimed to have perimenopause-related fatigue syndrome, and they were only recruited from one tertiary hospital in northern Taiwan. Thus, selection bias may limit the generalizability of these results of perimenopausal and postmenopausal women prone to fatigue syndrome. Second, since the validation study was conducted in one urban area (Taipei city), the language was limited to Mandarin, and, most importantly, these subjects were able to read, our sample may not be totally representative of all perimenopausal or postmenopausal women prone to fatigue syndrome from rural areas or other socioeconomically different areas. Third, we used a four-point Likert scale in this 25-question P-MFSMS to measure the variables of perimenopausal and postmenopausal women prone to fatigue syndrome, and so the measurement of severity by this scale may be relatively simple and rough. Fourth, certain items of the current 25-question P-MFSMS may make readers confused. For example, items from 18 to 20 in the section subtitled as “Slow down or adjust lifestyle” seemed to be similar. Additionally, the items in the “Frustration” subtitle section seemed to be absent of connection to “Have you used this method to deal with fatigue” and the answer “You feel helpless about fatigue”. However, based on the original “Chinese” words presented to the study subjects as well as the value of Cronbach’s α of the current P-MFSMS as 0.78, we expect that this scale will still be useful in Taiwanese women vulnerable to fatigue syndrome. However, we agree that all above-mentioned limitations contribute to the uncertainty of the reality of the 25-question P-MFSMS. Therefore, a large sample size to test the validity of the current scale will be used and we will continue to measure all parameters, such as α values each time the test is administered in the near future.

## 5. Conclusions

The results confirm that this scale has good reliability and validity, as well as conforms to the theoretical framework constructed by the results of qualitative research interviews, and thus can be promoted for clinical use. An effective treatment method for fatigue can provide perimenopausal women prone to fatigue syndrome with more coping strategies to overcome this syndrome. Medical staff should also actively offer relevant information, listen and empathize, gain recognition through understanding of perimenopausal women prone to fatigue syndrome, and, most importantly, provide effective tools, such as regular exercise, to help these women face their fatigue.

## Figures and Tables

**Table 1:** Qualitative categories based on grounded theory.

Qualitative Categories
1. Strive to maintain work energy and efficiency
2. Seek self-help from medical resources
3. Strive to maintain normal operation of the family
4. Make time for activities or exercise in busy life
5. Slow down or adjust lifestyle
6. Frustration

**Table 2:** The perimenopausal fatigue self-management scale (P-MFSMS). Participants were asked: “Have you used any of the following methods have to deal with your fatigue symptoms? If your answer is ‘yes,’ please provide how effective this method was.” The degree of the effectiveness was categorized as 25% effective = 1 point; 50% effective = 2 points; 75% effective = 3 points; 100% effective = 4 points.

Ways of Dealing with Fatigue	Method		Effectiveness			
	No	Yes	25%	50%	75%	100%
Strive to maintain work energy and efficiency						
1. You have temporarily left your work and not thought of other things to give yourself a break.	0	1	1	2	3	4
2. You have drunk coffee to refresh yourself.	0	1	1	2	3	4
3. You have kept yourself busy.	0	1	1	2	3	4
4. You have canceled a scheduled plan or arrangement.	0	1	1	2	3	4
5. You have consulted people who have gone through menopause.	0	1	1	2	3	4
Seek self-help from medical resources						
6. You have turned to traditional Chinese medicine to treat fatigue.	0	1	1	2	3	4
7. You have sought psychological counseling.	0	1	1	2	3	4
8. You have used Western medicine to supply hormone supplements.	0	1	1	2	3	4
9. You have used healthy foods or other alternative therapies.	0	1	1	2	3	4
Strive to maintain normal operation of the family						
10. You have asked your family to share the housework.	0	1	1	2	3	4
11. You have asked your family to help buy daily necessities.	0	1	1	2	3	4
12. You have relieved discomfort through a body massage and other methods.	0	1	1	2	3	4
Make time for activities or exercise in busy life						
13. You have found partners to participate in activities.	0	1	1	2	3	4
14. You have arranged simple and non-time-consuming activities, such as getting up and drinking water from time to time.	0	1	1	2	3	4
15. You have found time to exercise to increase your physical fitness.	0	1	1	2	3	4
16. You have listened to music to relax.	0	1	1	2	3	4
17. You have meditated or sat still.	0	1	1	2	3	4
Slow down or adjust lifestyle						
18. You have lived with these fatigue symptoms or discomfort.	0	1	1	2	3	4
19. You have endured fatigue.	0	1	1	2	3	4
20. You can bear these fatigue experiences.	0	1	1	2	3	4
21. For fatigue, you have adjusted your future lifestyle.	0	1	1	2	3	4
22. For fatigue, you have shared your management experiences with others.	0	1	1	2	3	4
Frustration						
23. You feel helpless about fatigue.	0	1	1	2	3	4
24. You feel angry about fatigue.	0	1	1	2	3	4
25. You have closed yourself to fatigue.	0	1	1	2	3	4

Notes: The right columns will need to be filled in if you have used the methods in the left columns and then fill in.

**Table 3:** Characteristics of the study subjects by categorical variables (*n* = 220).

Variables		*n*	(%)
Age (years)	51.28 ± 4.64		
Body mass index (kg/m^2^)	23.07 ± 3.04		
Marital status	Single	26	(11.8)
	Married	167	(75.9)
	Divorced	18	(8.2)
	Widowed	9	(4.1)
Education	Elementary school or below	4	(1.9)
	Junior high school	4	(1.8)
	Senior high school	60	(27.3)
	College or university	115	(52.3)
	Graduate school or above	37	(16.8)
Career	No paid salary	43	(19.6)
	With paid salary	176	(80.4)
Family type	Nuclear family	178	(81.3)
	Stem family	35	(16.0)
	Extended family	6	(2.7)
	None	212	(96.4)
	Yes	8	(3.6)
Menstruation status *	Perimenopause	99	(45.0)
	Menopause	111	(55.0)
Regular exercise **	None	141	(64.1)
	Yes	79	(35.9)
Chronic disease	None	167	(75.9)
	Hypertension	25	(11.4)
	Diabetes	13	(5.9)
	Renal disease	1	(0.5)
	Other	20	(9.1)

Data are presented as mean ± standard deviation or number (percentage). * Menstruation status: please refer to references [21,22,23]; ** regular exercise: please see the text and refer to [24].

**Table 4:** Distribution of the “perimenopausal fatigue self-management scale” and “perceived validity” (*n* = 220).

	(Yes)	25%	50%	75%	100%	M
Items in the Perimenopausal Fatigue Self-Management Scale	*n* (%)	*n* (%)	*n* (%)	*n* (%)	*n* (%)	(SD)
Strive for maintaining work energy and efficiency						
1. You have temporarily left your work and not thought of other things to give yourself a break.	173 (78.6)	40 (23.1)	68 (39.3)	50 (28.9)	15 (8.7)	2.2 (0.9)
2. You have drunk coffee to refresh yourself.	168 (76.4)	39 (23.2)	64 (38.1)	47 (28.0)	18 (10.7)	2.3 (0.9)
3. You have kept yourself busy.	116 (52.7)	41 (35.3)	33 (28.4)	38 (32.8)	4 (3.4)	2.0 (0.9)
4. You have canceled a scheduled plan or arrangement.	114 (51.8)	46 (40.4)	42 (36.8)	18 (15.8)	8 (7.0)	1.9 (0.9)
5. You have consulted people who have gone through the menopause.	102 (46.4)	49 (48.0)	33 (32.4)	17 (16.7)	3 (2.9)	1.8 (0.8)
Seek self-help from medical resources						
6. You have turned to traditional Chinese medicine to treat fatigue.	67 (30.5)	25 (37.3)	27 (40.3)	12 (17.9)	3 (4.5)	1.9 (0.9)
7. You have sought psychological counseling.	29 (13.2)	11 (37.9)	10 (34.5)	5 (17.2)	3 (10.3)	2.0 (1.0)
8. You have used Western medicine to supply hormonal supplements.	32 (14.5)	11 (34.4)	14 (43.8)	4 (12.5)	3 (9.4)	2.0 (0.9)
9. You haved used healthy foods or other alternative therapies.	106 (48.2)	45 (42.5)	38 (35.8)	21 (19.8)	2 (1.9)	1.8 (0.8)
Strive to maintain the normal operation of the family						
10. You have asked your family to share the housework.	146 (66.4)	43 (59.5)	48 (32.9)	40 (27.4)	15 (10.3)	2.2 (1.0)
11. You have asked your family to help buy daily necessities.	117 (53.2)	39 (33.3)	38 (32.5)	31 (26.5)	9 (7.7)	2.1 (1.0)
12. You have relieved discomfort through a body massage and other methods.	172 (78.2)	40 (23.3)	60 (34.9)	58 (33.7)	14 (8.1)	2.3 (0.9)
Make time for activities or exercise in busy life						
13. You have found partners to participate in activities.	128 (58.2)	24 (18.8)	48 (37.5)	42 (32.8)	14 (10.9)	2.4 (0.9)
14. You have arranged simple and non-time-consuming activities, such as getting up and drinking water from time to time.	151 (68.6)	55 (36.4)	49 (32.5)	4 (28.5)	4 (2.6)	2.0 (0.9)
15. You have found time to exercise to increase your physical fitness.	166 (75.5)	39 (23.5)	52 (31.3)	49 (29.5)	26 (15.7)	2.4 (1.0)
16. You have listened to music to relax.	182 (82.7)	38 (20.9)	61 (33.5)	52 (28.6)	31 (17.0)	2.4 (1.0)
17. You have meditated or sat still.	96 (43.6)	27 (28.1)	33 (34.4)	25 (26.0)	11 (11.5)	2.2 (1.0)
Slow down or adjust lifestyle						
18. You have lived with these fatigue symptoms or discomfort.	182 (82.7)	59 (32.4)	71 (39.0)	38 (20.9)	14 (7.7)	2.0 (0.9)
19. You have endured fatigue.	185 (84.1)	63 (34.1)	67 (36.2)	48 (25.9)	7 (3.8)	2.0 (0.9)
20. You can bear these fatigue experiences.	197 (89.5)	63 (32.0)	74 (37.6)	47 (23.9)	13 (6.6)	2.1 (0.9)
21. For fatigue, you have adjusted your future lifestyle.	178 (80.9)	49 (27.5)	70 (39.3)	48 (27.0)	11 (6.2)	2.1 (0.9)
22. For fatigue, you have shared your management experiences with others.	165 (75.0)	53 (32.1)	68 (41.2)	34 (20.6)	10 (6.1)	2.0 (0.9)
Frustration						
23. You feel helpless about fatigue.	82 (37.3)	41 (50.0)	28 (34.1)	10 (12.2)	3 (3.7)	1.7 (0.8)
24. You feel angry about fatigue.	72 (32.7)	38 (52.8)	22 (30.6)	11 (15.3)	1 (1.4)	1.7 (0.8)
25. You have closed yourself off to fatigue,	54 (24.5)	28 (51.9)	16 (29.6)	9 (16.7)	1 (1.9)	1.7 (0.8)

M, mean; SD, standard deviation.

**Table 5:** Differential item functioning (DIF) analysis.

Item	Person Class	Person Class	DIF Contrast	DIF SE	Rush–Welch	
					*t*	*p*-Value
m1	Perimenopause	Menopause	−0.18	0.12	−1.44	0.1509
m2	Perimenopause	Menopause	0.10	0.12	0.84	0.4022
m3	Perimenopause	Menopause	0.11	0.13	0.81	0.4178
m4	Perimenopause	Menopause	0.07	0.14	0.49	0.6233
m5	Perimenopause	Menopause	0.27	0.15	1.82	0.0704
m6	Perimenopause	Menopause	0	0.17	0	1.0000
m7	Perimenopause	Menopause	0.51	0.26	1.95	0.0527
m8	Perimenopause	Menopause	0.42	0.25	1.71	0.0898
m9	Perimenopause	Menopause	−0.19	0.14	−1.33	0.1847
m10	Perimenopause	Menopause	−0.13	0.13	−1	0.3192
m11	Perimenopause	Menopause	0.11	0.13	0.82	0.4112
m12	Perimenopause	Menopause	−0.15	0.12	−1.22	0.2254
m13	Perimenopause	Menopause	0	0.13	0	1.000
m14	Perimenopause	Menopause	−0.07	0.13	−0.56	0.573
m15	Perimenopause	Menopause	0.19	0.12	1.51	0.132
m16	Perimenopause	Menopause	0.05	0.13	0.38	0.705
m17	Perimenopause	Menopause	0.19	0.14	1.35	0.1787
m18	Perimenopause	Menopause	0.07	0.12	0.58	0.5622
m19	Perimenopause	Menopause	−0.18	0.12	−1.41	0.1609
m20	Perimenopause	Menopause	0	0.12	0	1.000
m21	Perimenopause	Menopause	−0.15	0.12	−1.2	0.2298
m22	Perimenopause	Menopause	−0.07	0.13	−0.56	0.5728
m23	Perimenopause	Menopause	−0.02	0.16	−0.13	0.9002
m24	Perimenopause	Menopause	−0.13	0.17	−0.74	0.463
m25	Perimenopause	Menopause	−0.18	0.19	−0.95	0.3418

SE: Standard error.

## Data Availability

Not applicable.

## Abbreviations

BMI	body mass index
CFS	chronic fatigue syndrome
CFI	Comparative Fit Index
CFA	confirmatory factor analysis
CVI	content validity index
DIF	differential item function
EFA	exploratory factor analysis
IFI	Incremental Fit Index
IRB	Institutional Review Board
KMO	Kaiser–Meyer–Olkin
MSA	measure of sampling adequacy
P-MFSMS	perimenopausal fatigue self-management scale
RMSEA	root mean square error of approximation
SD	standard deviation
SE	Standard error
TLI	Tucker–Lewis Index
